# Supplementary material for: Exploring the Role of Oleic Acid in Muscle Cell Differentiation: Mechanisms and Implications for Myogenesis and Metabolic Regulation in C2C12 Myoblasts
Source: Biomedicines. 2025 Jun 26;13(7):1568. doi: 10.3390/biomedicines13071568 (PMC12292418; doi:10.3390/biomedicines13071568)
Supplement: Supplementary file 1 [file biomedicines-13-01568-s001.zip › biomedicines-3704995-supplementary.pdf]

Table S1. Primers used in RT-qPCR analyses

Gene	Accesion numeber	Primer sequence	Amplicon size (bp)
MyoD	NM_010866.2	FOR: ggcagcgagcactacagtgg<br>REV: tcgacacagccgcactcttc	178
MyoG	NM_031189.2	FOR: cagcgccatccagtacattg<br>REV: accgaactccagtgcattgc	172
Murf	NM_001039048.2	For: catcattgacatctacaagc<br>Rev: ttttctcgtcttcgtgttcc	89
Mafbx	NM_026346.3	For: tcgcagccaagaagagaaag<br>Rev: aatcttctggaatccaggatg	181
Gapdh	NM_001289726.2	For: aaacctgccaagtatgatga<br>Rev: ctgttgctgtagccgtattc	217

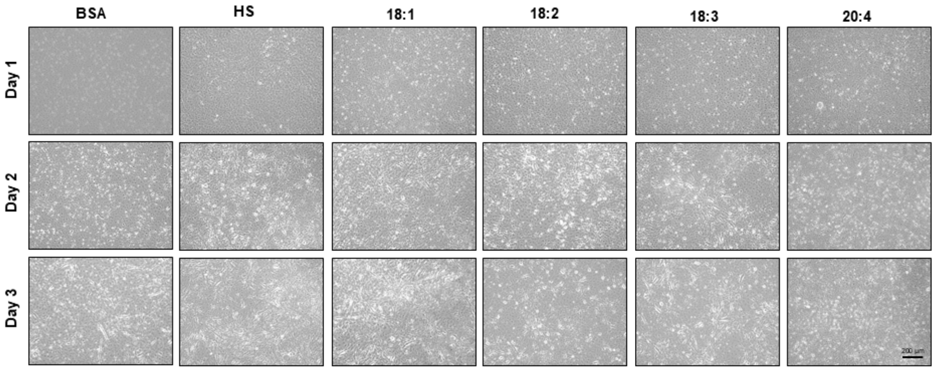

Figure S1. Time-dependent effects of fatty acids on C2C12 myogenic differentiation. Bright-field images (scale bar: 200 µm) of C2C12 myoblast monolayers cultured for 1, 2, or 3 days in the presence of BSA, 2% horse serum (HS), or 200 µM of the following fatty acids: oleic acid (C18:1), linoleic acid (C18:2), linolenic acid (C18:3), and arachidonic acid (C20:4).

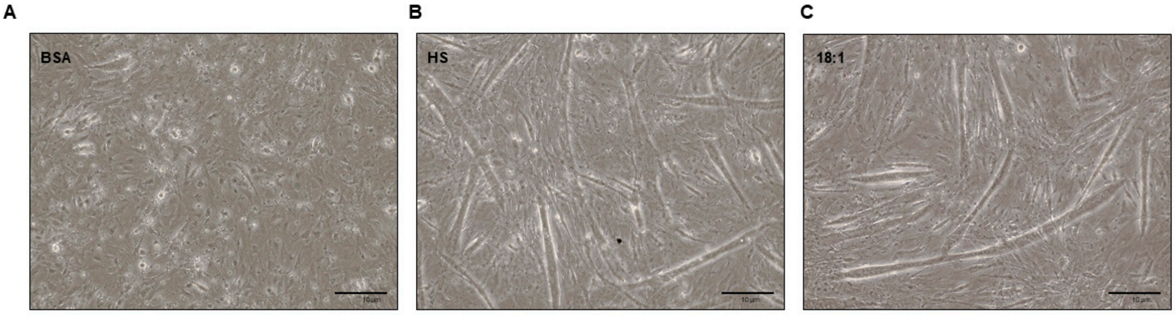

Figure S2. Effects of oleic acid on C2C12 myogenic differentiation and myotube formation. A: Bright field images (10 µm scale) of a monolayer of C2C12 myoblasts cultured for three days with BSA in the absence of FBS; **B**: differentiated C2C12 myotubes after three days in either 2% horse serum (HS), or **C**: 200 µM oleic acid (C18:1).
